# As above, so below: Deposition, modification, and reutilization of human remains at Marmoles cave (*Cueva de los Marmoles*: Southern Spain, 4000–1000 cal. BCE)

**DOI:** 10.1371/journal.pone.0291152

**Published:** 2023-09-20

**Authors:** Zita Laffranchi, Marco Milella, Juan Carlos Vera Rodríguez, María José Martínez Fernández, María Dolores Bretones García, Sylvia Alejandra Jiménez Brobeil, Julia Brünig, Inmaculada López Flores, Juan Antonio Cámara Serrano, Rafael M. Martínez Sánchez

**Affiliations:** 1 Department of Physical Anthropology, Institute of Forensic Medicine, University of Bern, Bern, Switzerland; 2 Departamento de Historia, Geografía y Antropología, Centro de Investigación en Patrimonio Histórico, Cultural y Natural, Facultad de Humanidades, Universidad de Huelva, Huelva, Spain; 3 Consejería de Turismo, Cultura y Deporte Delegación Territorial en Jaén, Junta de Andalucía Jaén, Spain; 4 Department of Legal Medicine, Toxicology and Physical Anthropology, University of Granada, Granada, Spain; 5 Department of Forensic Medicine, Institute of Forensic Medicine, University of Bern, Bern, Switzerland; 6 Independent Researcher, "*Arqueoantropología*", Sevilla, Spain; 7 Departamento de Prehistoria y Arqueología, Facultad de Filosofía y Letras, Universidad de Granada, Granada, Spain; 8 Departamento de Historia, Facultad de Filosofía y Letras, Universidad de Córdoba, Córdoba, Spain; University of California Santa Cruz, UNITED STATES

## Abstract

The deposition and manipulation of human remains in natural caves are well known for the Neolithic of Southern Iberia. The cultural meaning of these practices is however still largely unclear. Cueva de los Marmoles (CM, Priego-Córdoba) is one of the most important cave contexts from Southern Spain, which returned a large number of commingled skeletal remains suggesting its funerary use from the Neolithic to the Late Bronze Age. Here we discuss CM from a chronological and cultural perspective based on new radiocarbon, anthropological, and taphonomic analyses. These include the estimation of the minimum number of individuals, the exploration of fragmentation patterns characterizing different skeletal regions, and the macroscopic and microscopic analysis of modifications to the remains of possible anthropic origin. Radiocarbon data point to a funerary use of CM between the 5^th^ -2^nd^ millennium cal. BCE. MNI estimates reveal the presence of at least 12 individuals (seven adults and five nonadults). The low representation of elements from hands and feet suggests that individuals were placed in the cave while partially decomposed. Anthropic traces on the remains (e.g. fresh fractures, marrow canal modifications, and scraping marks) hint at their intentional fragmentation, cleaning from residual soft tissues, and in some cases reutilization. These practices are well-exemplified by the recovery of one "skull cup" and of two long bones used as tools. These data align with those from other cave contexts from the same geographic region, suggesting the presence, especially during the Neolithic period, of shared ideologies centered on the human body.

## Introduction

### Background

The use of caves for funerary purposes is a cultural phenomenon presenting a wide geographic and chronological distribution [1]. Examples of this practice include agrarian cultures from Europe [2, 3], Asia [4], Africa [5], the Americas [6] and the Pacific Islands [7]. Among the first Western Mediterranean farming societies, collective burials are often found in natural caves, hypogea, and megalithic chambers [8]. Indeed, it is with the early Neolithic (around the 6^th^ millennium BCE) that true necropolises in natural caves begin to represent a widespread cultural phenomenon in this area, with different examples being well known in the Italian peninsula and Sicily, Sardinia, and France [9–11]. For the Iberian Peninsula, the archaeological traces of funerary use of caves, although already documented for the Early Neolithic, become particularly frequent starting from the late Neolithic (5^th^-4^th^ millennium BCE) in modern-day Portugal (Algar de Bon Santo) and Spain (Valencian Community, Central Meseta, and, especially, Andalusia) [12–16].

This chronological trend characterizes, for example, Andalusia (regional focus of the present contribution), where disarticulated bone deposits appear in parallel with primary interments during the Early Neolithic (2^nd^ half of 6^th^ millennium BCE) [17, 18]. In some cases, these finds feature clear traces of manipulation and defleshing, often interpreted as the result of consumption processes [19, 20]. Here, as in general in Southern Iberia, the use of burial caves becomes more frequent from the 4^th^ millennium onwards [21]. This trend is parallel to the use of megalithic chambers and hypogea [8, 22], the latter becoming particularly recurrent throughout the 3^rd^ millennium cal BCE [23]. Examples of cave contexts from Andalusia include the *Cueva de los 40* (also in Priego de Córdoba), dated between 3700–2900 cal. BCE [16], and returning a commingled assemblage of skeletal remains of up to 41 individuals, and the cave of *Camino del Molino* (Caravaca, Murcia, 2800–2200 cal. BCE), including the remains of ca. 1300 individuals [24]. Occasionally, the deposits of human remains in karstic environments persist until the 2^nd^ millennium cal. BCE [25, 26] as a repetition of previous burial modalities, similarly to the continued use of megalithic chambers for funerary purposes [27].

### Archaeological context and history of finds

**Fig 1:**
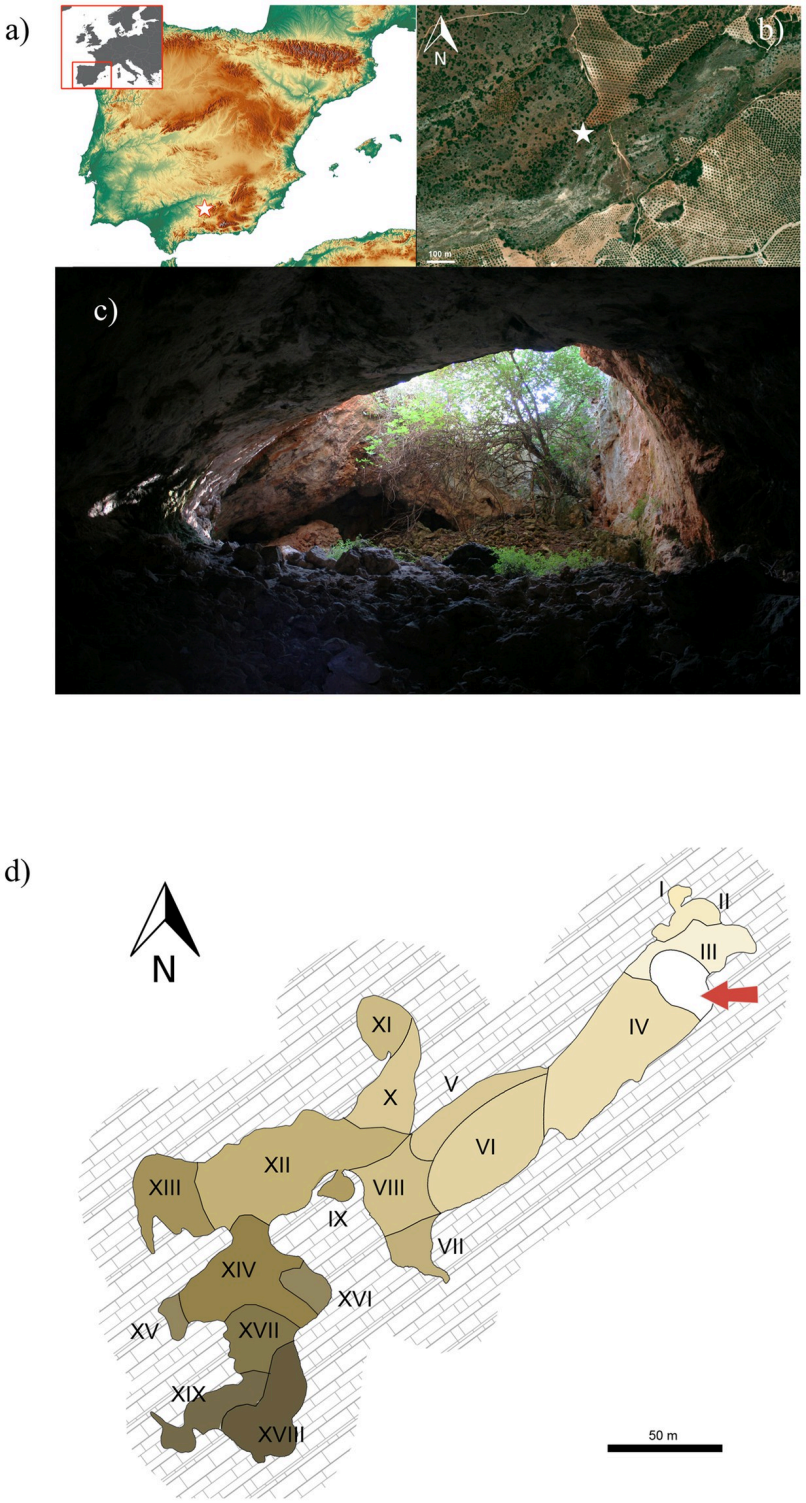
a-b) Geographical location of Marmoles cave; c) entrance to the cave; d) distribution of cave zones (The map of Spain and the orthophotography have been edited by R.M. Martínez Sánchez based on freely available material from the National Geographic Institute of Spain (IGN). Map of Europe elaborated by R.M. Martínez Sánchez based on images freely available from Wikimedia Commons; Photograph of cave by J.C. Vera Rodríguez and cave plan adapted from [29] by R.M. Martínez Sánchez and M. Milella).

*Cueva de los Mármoles* (Priego de Córdoba, henceforth CM) is a natural cave of hydrological origin located in the Sierra de los Judíos, to the east of the Sierras Subbéticas Cordobesas (Fig 1a and 1b). This area is well known for intense prehistoric occupation in open-air and cave sites, mainly dating to the Early and Late Neolithic, Copper, and Bronze Age [28]. Located at an altitude of about 900 m, the cave has a wide entrance (Fig 1c) composed of a large sinkhole (diameter: ca. 10 m: depth: ca. 8 m) proceeding with a slight vertical inclination (-45.27 m). With an extension of more than 370 m, it is one of the largest caves in the province of Córdoba.

Topographically, the cave (Fig 1d) is represented by a wide, descending gallery (*Rampa*) which then proceeds horizontally at the bottom (*Sala de los Murciélagos*). A large, final room (*Gran Salón*) opens to the left and includes a chaotic amount of stone blocks. These main spaces include secondary rooms of smaller dimensions, e.g. *Sala de las Columnas*, *Sala de los Nichos* and *Sala del Pánico*. For descriptive purposes, the cave can be spatially subdivided into nineteen zones. Zones III and IV are near the cave entrance and are reached by sunlight for most of the day. Light (although weak) also reaches Zones II, VI and VIII, while the rest of the cave is completely dark.

The first archaeological excavations at CM date to 1934 [30] and returned finds broadly assigned to the Late Neolithic and Early Copper Age based on typological criteria, as well as the first interpretation of the cave as a funerary chamber based on the numerous human remains visible on the surface.

Starting from the early 1960s [31, 32], the cave became one of the most targeted archaeological sites in the region by amateur and clandestine excavators. The material extracted in this way (e.g. ceramic artefacts, ornaments, and lithic tools) passed through various private collections, to be then incorporated into the Municipal Historical Museum of Priego of Córdoba. Some of these finds, interpreted as Neolithic, were published toward the end of the 1970s [33].

Between 1982 and 1987 the cave was explored by six different archaeological campaigns mainly targeting the cave entrance [34]. These investigations revealed an Early Neolithic occupation (Middle Andalusian Neolithic according to the standards of the late 20^th^ century) characterized by the presence of incised, impressed, and “a la Almagra”-red slip decorated ceramics. Associated with this phase were the remains of a sunken structure featuring clay floors and at least two post holes containing a large number of charred cereals [35]. The latter were more recently radiocarbon dated to the last quarter of the 6^th^ millennium cal. BCE [36]. In addition to the Neolithic levels, the excavation highlighted an underlying stratigraphic package preliminarily dated to the Middle-Upper Palaeolithic [37]. Different surface findings (mainly pottery) also point to a parallel non-funerary use of the cave during the Late Neolithic, Copper Age, Bronze Age [29, 38] and Andalusian Middle Ages (Caliphate of Córdoba, Umayyad culture, 10^th^-11^th^ century AD) [29].

The most recent archaeological work at the site was directed in 2018 by some of the authors. It included, besides a detailed survey, a re-excavation of the sectors explored in the 1980s and the systematic sampling of sediments for archaeobotanical analyses. This work led to the completion of the osteological collection from the site as well as to the confirmation of previously suggested periodization of the occupation levels.

It is important to stress that the human remains included in this study are not stratigraphically assigned, but are surface finds from the interior of the cave. The remains were mainly collected during the surveys of 1998 [29] and 2018, as well as, in some cases, by speleologists and other researchers in previous years. For this reason, we considered only those finds unequivocally assigned to specific cave zones.

### Aims

As already mentioned, archaeological works at the cave returned over the years a large number of commingled human skeletal remains. Of these, a select number of elements has been the focus of published reports due to their possible prehistoric chronology and presence of traces of manipulation [39–41]. Of particular interest is an adult calvarium reworked as a "skull cup" and a tibial shaft apparently used as a tool. However, no study has been carried out on all of the available anthropological remains from the cave. Accordingly, no estimates are available on the possible number and demographic distribution of the deposited individuals or on the overall patterns of anthropic traces in the sample. Consequently, the natural and anthropic processes responsible for the skeletal assemblage of CM, and their cultural and behavioral meaning are still largely unexplored.

These considerations prompted an extensive macroscopic and microscopic analysis of the human remains retrieved from the cave over the years, as well as a new ^14^C study with the aims of:

1. Providing new insights about the funerary use of the cave (e.g. frequency of depositions and possible selective practices based on sex and/or age-at-death) by means of an estimate of the minimum number of individuals (MNI) and their demographic distribution (age-at-death and sex).
2. Evaluating the actual presence and type of anthropic modifications to the human remains based on the assessment of their anatomical preservation, spatial distribution, as well as their macroscopic and microscopic analysis.
3. Contextualizing chronologically the use of the cave by means of new radiocarbon dates.

## Materials and methods

### Radiocarbon analysis

For the ^14^C analysis we selected seven bone samples representing zones II, III, IV, VIII, XII, and XVI (S1 Table). Six samples were analyzed at the CNA laboratory in Seville (Spain) and one (Beta-645979) at Beta Analytic (Miami, USA). All samples were subjected to collagen extraction and purification with alkali and ultrafiltration, according to internal protocols. The conventional results were calibrated using the terrestrial atmospheric curve IntCal20 [42], in Oxcal, version 4.4. [43]. The same software was used to perform a Bayesian model in order to evaluate the presence of distinct depositional phases and hiatuses based on a sequential hypothesis of phases [43].

### MNI

In this study we applied the protocol of Mack et al. [44], which is adapted from the zonation method of Knüsel and Outram [45]. We first checked for possible conjoining of fragments based on anatomical fitting and/or quantitative (e.g. size and shape) or qualitative (e.g. coloration) criteria. We considered conjoined fragments as a single element in the following analysis. We scored each element (i.e. fragment) according to the presence or absence (coded as 1 and 0, respectively) of a set of landmarks (i.e. anatomical features) specific to each bone (for the full list and details of the landmarks see Supplementary Material in [44]). We considered a landmark as "present" only if it was more than 50% preserved. In order to have a more complete view, we also counted and registered the fragments whose landmark’s expression was below the 50% threshold but for which the anatomical attribution was still possible (see column "fragm <50%" in S2 Table). After this preliminary stage, the analysis included the following steps:

1. we first examined morphologically all fragments trying to allocate them to specific bones and sides. For each fragment, we also tried to estimate the age-at-death and sex of the individual. In most of the cases, only a rough determination of the age-at-death was possible. We therefore opted for using only two classes, separating adults (≥ 20 years old) from nonadults (< 20 years old). This subdivision was based on the degree of epiphyseal fusion and/or diaphyseal length [46, 47], stage of dental development [48, 49], and degree of wear of the crown [50]. We attempted to determine sex for cranial, mandibular, and coxal fragments, based on the evaluation of dimorphic features of these skeletal elements [51, and methods listed in 52].
2. we then sorted the resulting dataset by element and further divided it by side and age class (adult/ nonadult). After calculating the total number of observations for each landmark (MNE: minimum number of elements), or, in the case of dental remains, tooth type and side, the highest value from each side corresponds to the MNI.
3. we calculated MNI separately on adult and nonadult remains. We decided to calculate a single MNI without subdividing this analysis by cave zone to avoid the possible bias in the spatial distribution of the remains resulting from postdepositional factors (e.g. ancient and modern human and faunal activity). For the fragmentation analysis, however (see below), we also included separated analyses by cave zones.

### Fragmentation analysis

Intra-skeletal patterns of bone fragmentation and preservation are useful when discussing the type of funerary treatment (e.g. primary *vs*. secondary deposition) of human remains in a given context. Especially for cave finds like those of CM, the combined analysis of fragmentation and preservation patterns and the presence and distribution of bone lesions of possible anthropic origin provide an ideal angle for developing any biocultural interpretation [11]. Following these criteria, in this work we calculated for each bone the following indices:

1) the "Percentage completeness" [cf. 53], i.e. the average number of landmarks recorded as "present" on each identified element divided by the maximum number of landmarks possible for that bone element.
2) the Fragmentation index (FI), which is the ratio between the number of identified specimens (NISP) of a determinate element (bone) by the minimum number of that element (MNE) [54].

We calculated the Percentage completeness and FI first for the total sample and second for each cave zone. After having verified the deviation of these variables from a normal distribution through a Shapiro-Wilk test, we then tested for statistically significant differences between zones by means of Kruskal Wallis and Steel Dwass tests, which are nonparametric alternatives to one-way ANOVA and Tuckey tests, respectively. We then applied a Spearman test to assess the correlation between Fragmentation Index and Percentage Completeness. Following the suggestion of Mack et al. [44], we also excluded from these steps the sternum, ribs, and vertebrae due to difficulties in addressing the causes of fragmentation for these fragile bones [e.g. decomposition vs taphonomic agents–see also 44].

3) the Representation Index, i.e. the ratio between the observed and expected MNE based on the MNI [see 11].

These three indices provide different types of information: Percentage Completeness measures the overall preservation of a specific element, independently from their degree of fragmentation, the latter being indeed described by FI [cf. 44]. The Representation Index is especially useful when exploring differential patterns of preservation between skeletal regions, and discussing their possible taphonomic vs. anthropic origin (e.g. secondary funerary practices, retrieval of skeletal element) [11].

We performed all statistical analyses in JMP^®^, Version 17.0.0. (SAS Institute Inc., Cary, NC, 1989–2022), setting alpha at 0.05.

### Bone modification

We analyzed each fragment macroscopically and microscopically (Dinolite USB digital microscope), and annotated the presence, location, and main features of surface modifications of possible anthropic origin. These were classified following the criteria of Bello et al. [55], Greenfield et al. [56], and White [57]. We considered the following types of modifications: cutmarks (slicing and scrape marks) [sensu 55–58], chop marks, and fresh fractures. In addition, we also checked for the presence of possible traces of intentional alteration of the medullary canal [59]. The latter results in the smoothing of the canal surfaces and, in better-preserved shaft fragments, tubular cavities. These traces have been associated with the extraction of marrow from bones [59].

We recorded the anatomical distribution of each feature using the landmarks proposed be Mack et al. [44]. In eight cases (MR-18-Z223, MR-98-65, MR-18-Z40, MR-18-Z76, MR-18-Z107, MR-18-Z126, MR-18-Z135 and MR-18-Z223) the lesion could not be assigned to one of the landmarks and was recorded on the landmark closest to the affected area.

For a subset of lesions we also performed high precision dental silicone casts (Provil Novo Light), which we then analyzed further with a digital microscope (Keyence VHX-5000 equipped with VH-Z20P and RZx20-x200 lenses).

We supplemented the macroscopic and microscopic analyses of the "skull cup" by implementing a 3D photogrammetric model using 3DF Zephyr (version 7.009), and by performing a computer-assisted tomographic study (henceforth CT). The aims of the latter are to clarify the nature of the perforating lesion on the left parietal (see above) and obtain a three-dimensional documentation of the remain. The tomographic study was performed at University of Córdoba, Spain, using a 32-slice helical scanner (Revolution ACT; General Electric Health Care, Milwakee, Wiscosin, US). Scan settings include a slice thickness of 0.6 mm, collimator pitch 0,75, X-ray tube potential 120 kVp, tube current exposure time 120 mAs, and matrix 512–512 reconstructed with bone window. CT data were further visualized in 3D Slicer (v. 5.2.2, www.slicer.org).

## Results

### Chronology (^14^C)

**Fig 2:**
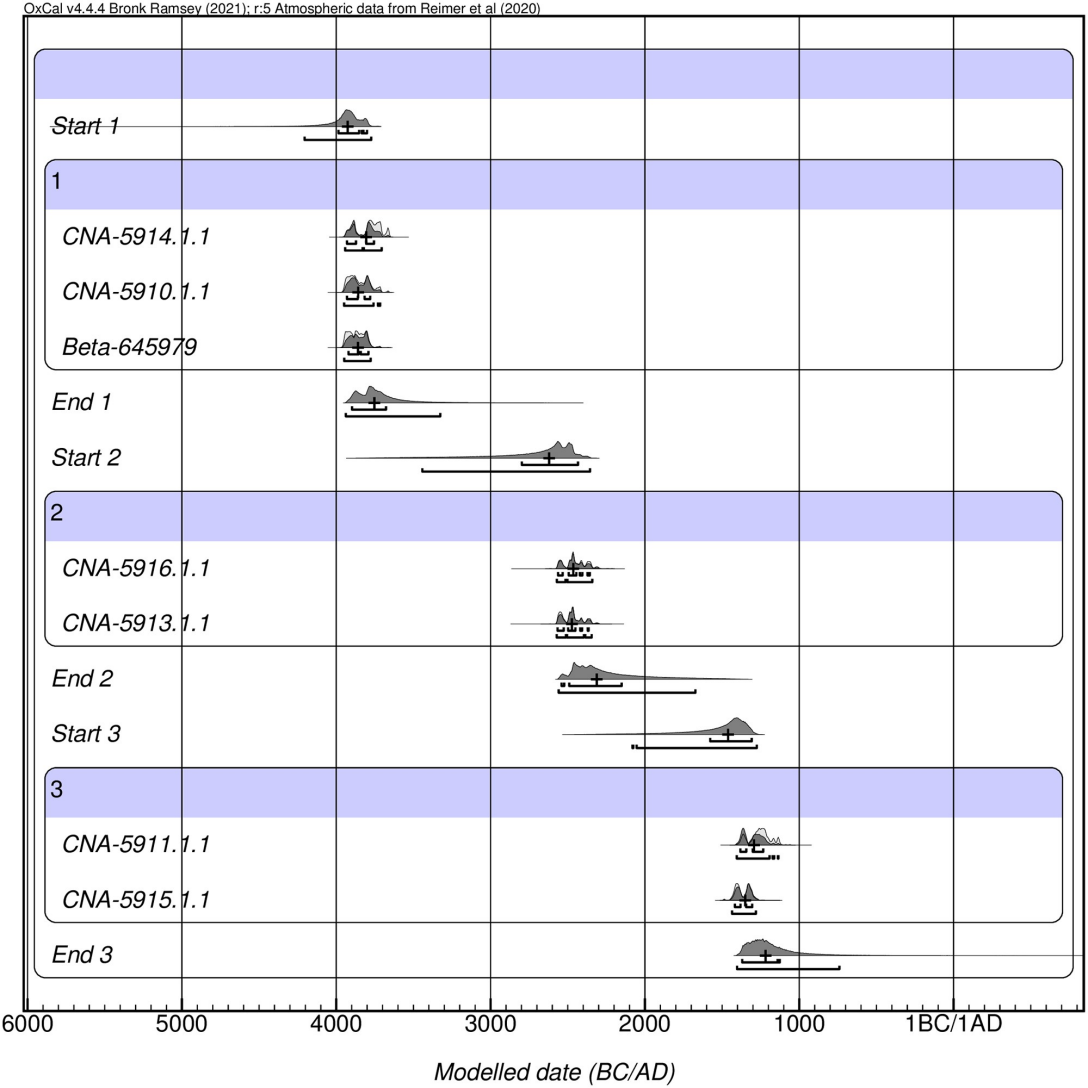
Result of the Bayesian modeling of the radiocarbon dates from Marmoles.

All dates fall between the end of the 5^th^ millennium and the second half of the 2^nd^ millennium cal. BCE (S1 Table). These results contradict previous suggestions about a recent origin of these finds [38], but they post-date the Early Neolithic occupations documented by archaeological excavation [36]. Moreover, the obtained dates tend to cluster around distinct chronological phases. The inclusion of these data into a Bayesian model with three phases separated by hiatuses results in an agreement index (Amodel) of 92.6%, well beyond the accepted threshold of 60% and reflecting a high internal consistency [60]. This model depicts a temporal sequence of three phases, separated from each other by two hiatuses without evidence of deposits and occupying approximately one thousand years (Fig 2). The first phase, documented by three radiocarbon dates from zones II, IV, and XVI (CNA-5914.1.1: 5000 ± 35 BP; CNA-5910.1.1: 5040 ± 35 BP, and Beta-645979: 5060 ± 30 BP), corresponds to the first quarter of the 4^th^ millennium cal. BCE (3900–3750 cal. BCE). After a hiatus between 3700 and 2600 cal. BCE, a second depositional phase would occupy the range 2600–2300 cal. BCE, as reflected by samples CNA-5916.1.1 (3950 ± 35 BP) and CNA-5913.1.1 (3960 ± 35 BP) from zones VIII and XII, respectively. After this, and another hiatus between 2300 and 1400 BCE, the last depositional phase occupies the time range between 1400 and 1200 cal. BCE, represented by the samples CNA-5911.1.1.1 (3010 ± 35 BP) and CNA-5915.1.1 (3110 ± 30 BP), both from zone III.

### MNI

The skeletal sample from CM amounts to a commingled set of 411 bone fragments and 10 loose teeth (S2 Table). An additional 47 teeth are still attached to the relative jaw fragments. With the exception of four bone fragments, we managed to anatomically identify all finds. Fragmentation hampered, in some cases, assessments of the side of the fragments. This led to the siding of 271 out of 312 (86.9%) bilateral bones fragments (e.g. parietal bones, long bones, and coxal bones). Cranial bones are the most frequent specimens (65/406: 16.0% of anatomically identified fragments) followed by the femora (55, 13.5%). With just one fragment each, the sternum, patella, and foot phalanges are the least represented bones (Table 1; S1 Fig).

**Table 1:** Distribution of human remains by skeletal region/bone.

Skeletal elements/bones	N	%
Cranium	65	16.0
Cervical vertebrae	10	2.5
Clavicle	7	1.7
Coxal bones	26	6.4
Fibula	15	3.7
Foot phalanges	1	0.2
Hand phalanges	4	1.0
Humerus	39	9.6
Lumbar vertebrae	16	3.9
Mandible	10	2.5
Sternum	1	0.2
Femur	55	13.5
Metacarpal bones	3	0.7
Metatarsal bones	3	0.7
Patella	1	0.2
Radius	8	2.0
Ribs	47	11.5
Sacrum	11	2.7
Scapula	14	3.4
Tarsal Bones	7	1.7
Thoracic vertebrae	21	5.2
Tibia	31	7.6
Ulna	12	2.9

We observed traces of carnivore activity and rodent gnawing on 13 fragments (3.2% of total).

As mentioned in the method section, the calculation of the MNI was based on those finds preserving the landmarks proposed by Mack et al. [44]. Based on this criterion, our calculation considers 340 bone fragments and 57 dental remains.

For adults the largest number (7) is returned by the right humeral landmark 5 (capitulum) and the right tibial landmark 6 (distal shaft). For nonadults the left femoral landmark 6 (gluteal tuberosity) provides the largest estimate (5). Trying to subdivide dental remains between adults and nonadults using tooth developmental and crown wear stages provides the same adult estimate (7, based on LRM1), but a lower number for nonadults (MNI: 1). Among the nonadults we could identify the presence of 3 individuals between 3–12 years old ("Child" category) and 2 adolescents (13–20 years) according to the age categorization of Buikstra and Ubelaker [52].

We tried to calculate the sex ratio of adults using the MNI returned from the sexually dimorphic landmarks of the pelvis and skull (e.g. greater sciatic notch, mastoid process, occipital protuberance). This led to an estimate of four females and one male (based on the right *os coxae* landmark n. 7: "greater sciatic notch"). By observing on the same iliac fragments the degenerative changes of the auricular surface [61], we propose that the females included two young adults and one old individual (YA: 20–35 years and OA:>50 years), whereas the male was likely aged a middle adult individual (MA: 35–50 years old).

Summing up these observations, we can propose a MNI of 12, including seven adults and five nonadults. Adult individuals include four females (two YA, one OA, and two individuals for which only a generic adult age (i.e. > 19 years old) was possible), two males (one MA, one adult), and one adult whose sex was not assessable.

### Fragmentation analysis

The talus, calcaneus, metacarpals, and metatarsals show the highest Percentage completeness (between 80–100%—S3 Table). The most complete long bone is the radius (85.7% and 64.2% for the left and right respectively–for a total of 85%).

The cranium presents the lowest completeness (2.7%) and the highest level of fragmentation (FI = 8.1) (S4 Table).

The Representation Index (Table 2, S2 Fig) reflects unsurprising differences between skeletal regions and age groups. In adults, the atlas, axis, clavicle, talus and calcaneus, metatarsals, and metacarpals show a representation below 50%. Conversely, the sacrum, humerus, and cranium in this age class are the most commonly represented elements (85.7%-100%). Nonadults show, in general, a worse skeletal representation, and a complete absence of the atlas, radius, talus, and metacarpals. The best-represented bones in nonadults are the humerus, femur, and tibia, all presenting representation index values above 60%.

**Table 2:** Representation Index of skeletal elements/bones for adults and nonadults. The index is calculated using 12 as total MNI and 7 and 5 as MNI for adults and for nonadults respectively. RIs of bilateral bones are presented for simplicity as single value. MNE for paired bones are obtained from the sum of left and right MNEs.

Bone/Element	MNE*	Total	Rindex (%)	Adults			Nonadults		
		expected		MNE*	expected	Rindex (%)	MNE*	expected	Rindex (%)
Cranium	8	12	66.7	6	7	85.7	2	5	40.0
Mandible	6	12	50.0	5	7	71.4	1	5	20.0
Atlas	2	12	16.7	1	7	10.0	1	5	20.0
Axis	2	12	16.7	1	7	10.0	1	5	20.0
Clavicle	6	24	25.0	4	14	28.6	2	10	20.0
Humerus	21	24	87.5	13	14	92.9	8	10	80.0
Scapula	10	24	41.7	9	14	64.3	1	10	10.0
Radius	7	24	29.2	7	14	50.0	0	10	0.0
Ulna	9	24	37.5	8	14	57.1	1	10	10.0
Metacarpals	3	120	2.5	3	70	4.3	0	50	0.0
Os coxae	10	24	41.7	8	14	57.1	2	10	20.0
Sacrum	8	12	66.7	7	7	100.0	1	5	20.0
Fibula	10	24	41.7	9	14	64.3	1	10	10.0
Calcaneus	4	24	16.7	3	14	21.4	1	10	10.0
Talus	2	24	8.3	2	14	14.3	0	10	0.0
Tibia	17	24	70.8	10	14	71.4	7	10	70.0
Femur	17	24	70.8	10	14	71.4	7	10	70.0
Metatarsal	3	120	2.5	2	70	2.9	1	50	2.0

As expected, completeness and fragmentation are inversely correlated, i.e. bones showing the higher fragmentation are on average the less complete ones (Spearman rho = -0.4; p<0.0001).

Skeletal remains show a heterogeneous spatial distribution, with zones II, III, VI, X, and XII contributing, in total, to 71.3% of the sample (293/411). Zones I, IX, XV, XVII, and XIX returned no remains (see Table 3 for details and Fig 1d for the position of each zone in the cave).

**Table 3:** Distribution of human skeletal and dental remains among cave areas. Only fragments clearly assigned to zones are considered. This number also includes fragments with no available landmarks.

Cave Zone	n remains	%
I	0	0.0
II	59	14.3
III	50	12.1
IV	29	7.0
V	17	4.1
VI	53	12.9
VII	20	4.9
VIII	12	2.9
IX	0	0.0
X	58	14.1
XI	19	4.6
XII	73	17.7
XIII	14	3.4
XIV	1	0.2
XV	0	0.0
XVI	3	0.7
XVII	0	0.0
XVIII	4	1.0
XIX	0	0.0

The Kruskall Wallis tests on Percentage Completeness and Fragmentation index show no statistically significant differences between zones for these variables (Percentage Completeness: ChiSquare = 19.1649; DF = 13; p = 0.1181; FI: ChiSquare = 21.4401; DF = 13; p = 0.0647). In general, the cranium, humerus, os coxae, femur, and tibia are the most represented skeletal elements in each zone (S3 Fig). From a demographic point of view, adults and nonadults are often represented in the same zones with the exception of zones V, VIII, XI, and XVIII, which returned only adult remains (S2 Table). Given the small sample size and the possible error associated with sexing isolated fragments, we did not calculate zone-specific sex ratios.

### Bone modifications

#### General patterns

130 out of 411 skeletal fragments (31.6%) show at least one lesion. The number of traces per fragment ranges from 1 (82 fragments) to 5 (1 fragment). In all of these cases, the coloration and shape of the lesion suggest old events, and the morphological features of the lesions point to a chronological timing when the bone was still "fresh", i.e. relatively flexible. S5 Table shows the calculated minimum number of skeletal elements presenting lesions subdivided by bone/element, side, and age category. As mentioned in the method section, we consider the following features: fresh fractures (*f)*, cut marks (including scraping marks) (*c)*, chop marks (*ch)*, and alterations of the medullary canal (*malt)*. Like all calculations in this work, this count includes only those elements whose landmarks and side were identified (the latter in the case of bilateral bones). However out of thoroughness S6 Table also presents the same calculation for all available fragments (independently from landmark preservation and side identification).

**Fig 3:**
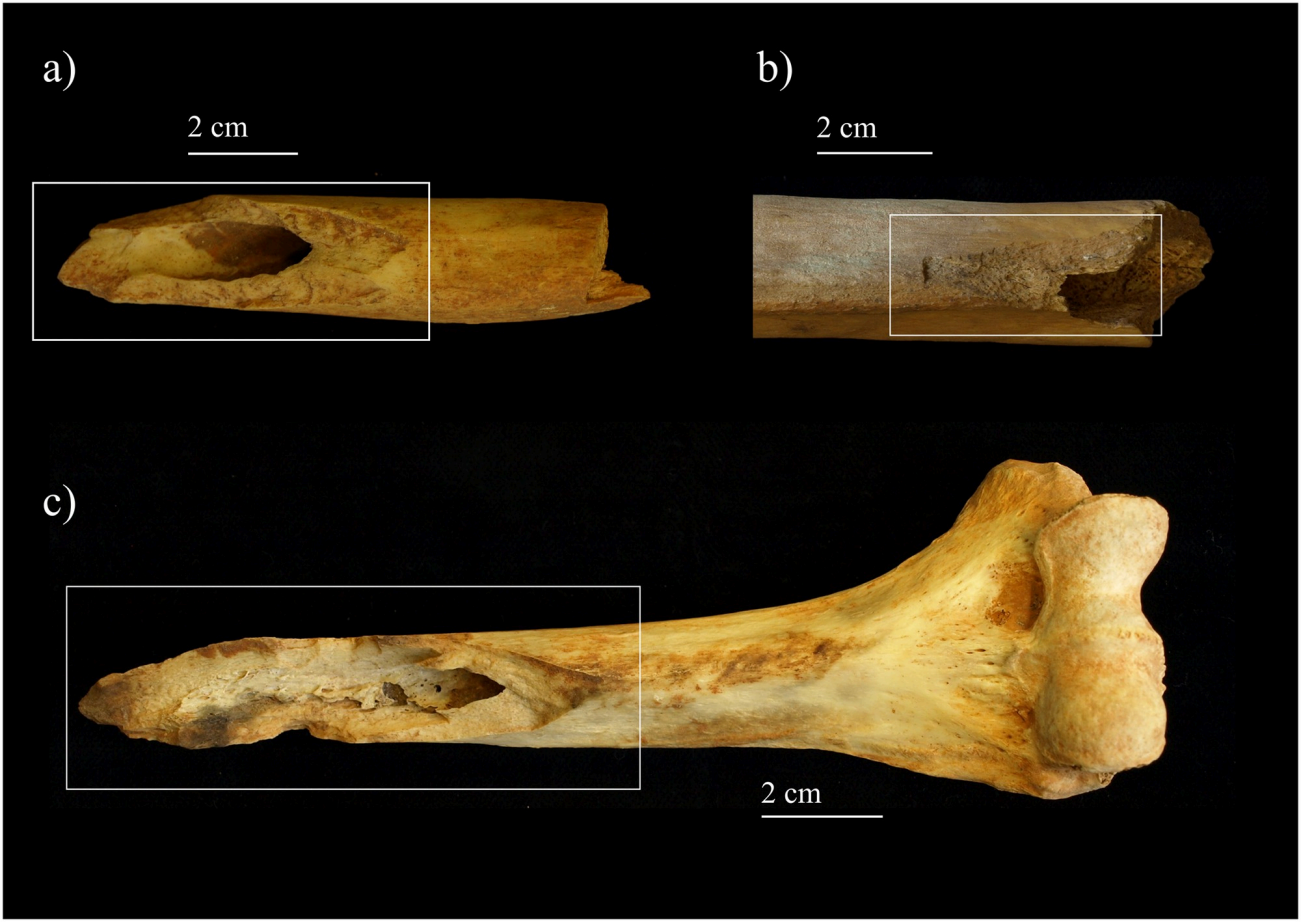
Examples of fresh fractures from Marmoles cave: (a) right humerus; (b) left femur; (c) right humerus (photographs by Z. Laffranchi).

Fresh fractures are the most frequent type of lesion at CM, and are particularly found in the cranium and long bones (Fig 3).

**Fig 4:**
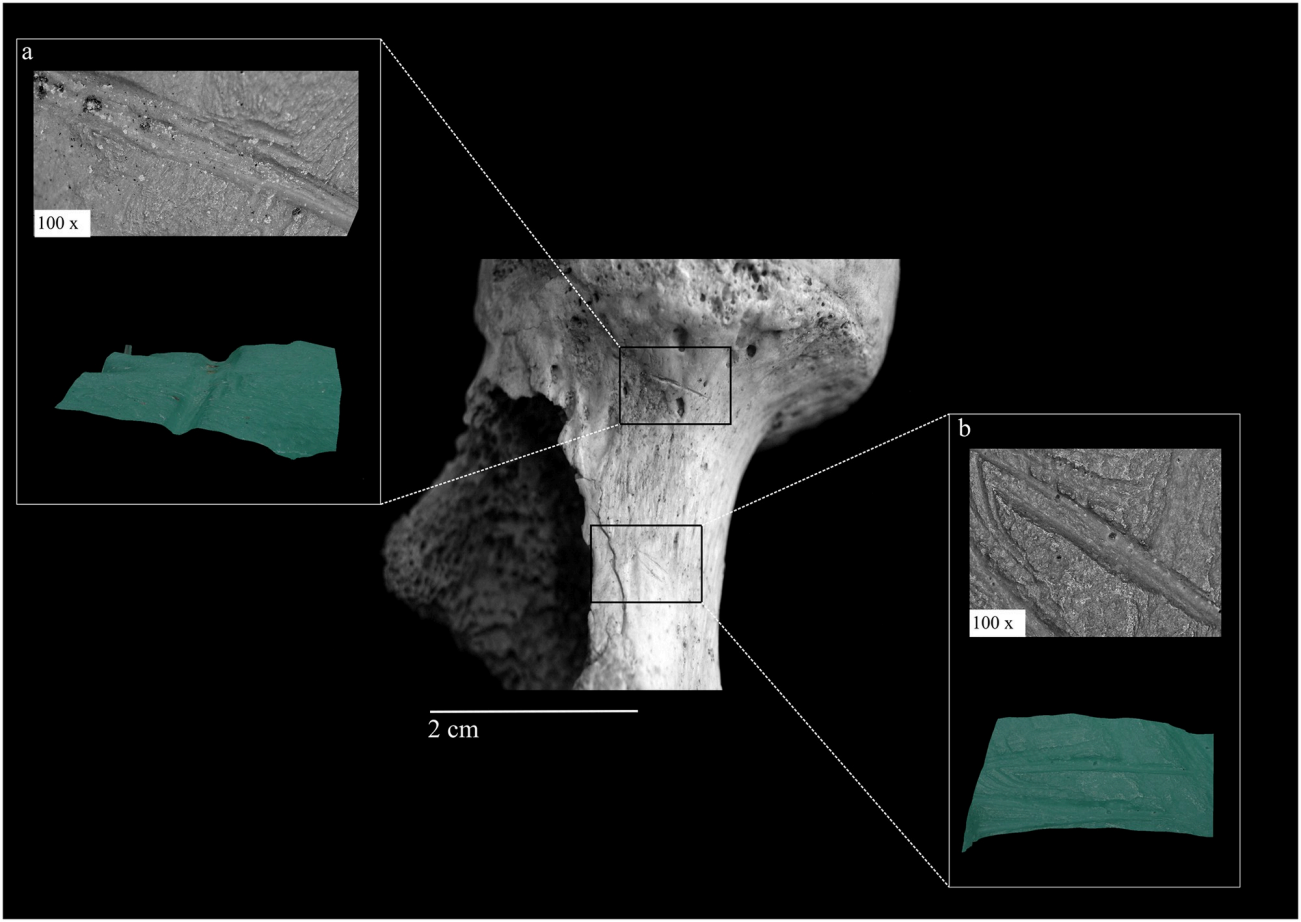
Cutmarks on the anterior side of a right femur: location on the bone, microscopic appearance and 3D profile of the lesion obtained from the digital microscope analysis (a, b) (photographs by Z. Laffranchi, digital microscope images captured by J. Brünig).

Cut and chop marks are less frequent, having been observed on seven and four fragments, respectively, including fragments from the mandible, cranium, fibula, humerus, and femur (cut marks–Fig 4) and os coxae, tibia, humerus, and femur (chop marks) (S5 and S6 Tables).

**Fig 5:**
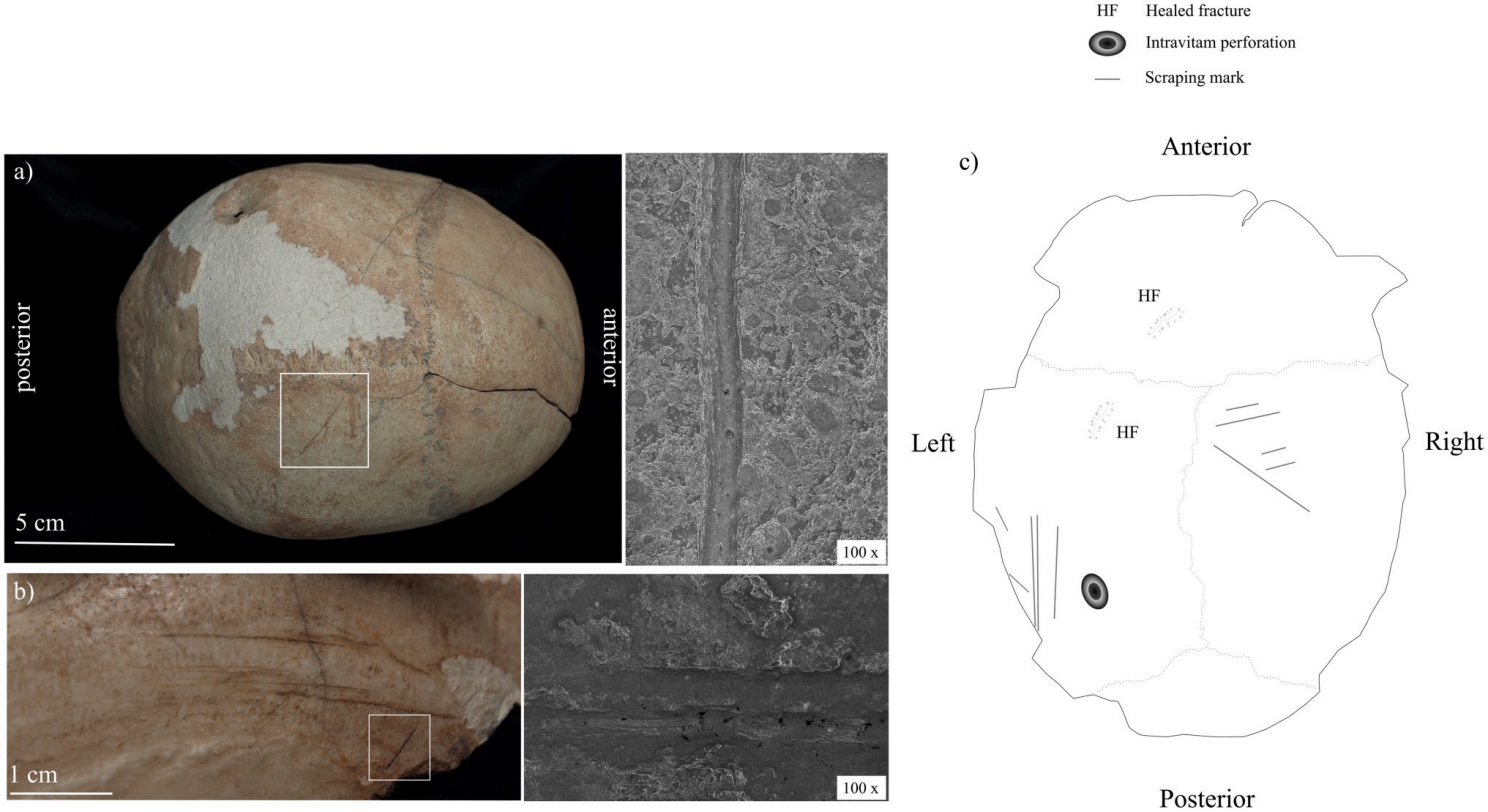
Examples of scraping marks on the skull cup MA 220: Macroscopic and microscopic appearance. (a) right parietal; (b) left parietal; (c) schematic representation of ectocranial surface (photographs by Z. Laffranchi; drawing by M. Milella).

Scraping marks appear on at least one cranial remain, the "skull cup" MA-220 (Fig 5), and alterations of the medullary canal characterize 17 long bone fragments (4 femoral, 7 tibial, and 6 humeral), which are always present in conjunction with fresh fractures (S5 and S6 Tables). The microscopic analysis of cut marks highlights relatively broad, asymmetric, and uneven profiles (S4 Fig) and the presence of internal microstriations. All of these features fit previous microscopic descriptions of stone tools cut marks [58, 62, 63]. The shallow profile of the marks on the skull cup MA-220 substantiate their macroscopic attribution to scraping action.

#### Specific cases

In this section we provide a detailed description of two specimens (MA-220 and MA-213-1187) selected due to their possible biocultural relevance.

**Fig 6:**
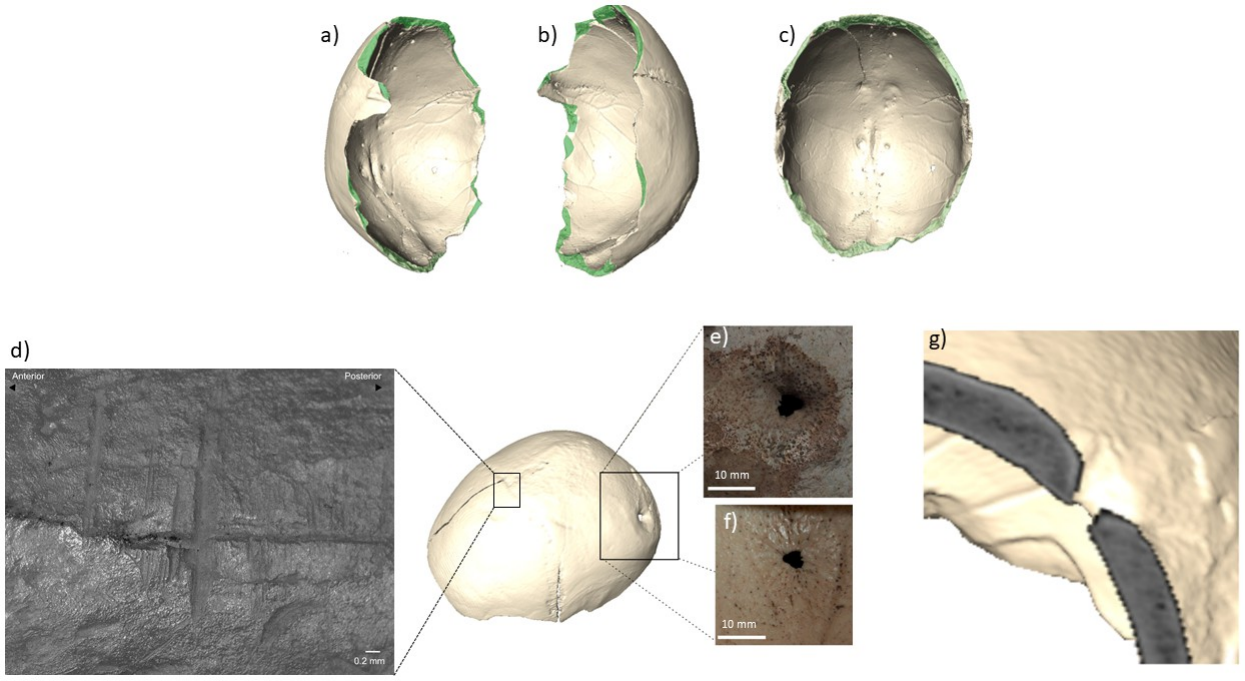
"Skull-cup" MA-220: right (a), left (b), and inferior (c) view showing the percussion marks (green areas) along the rim of the cranial vault; microscopic view of scraping marks near the bregmatic area (d); exocranial (e) and endocranial (f) views of the circular defect on the left parietal bone; (g) computed tomographic image of the left parietal bone at the level of the discontinuity (photographs by Z. Laffranchi, CT images elaborated by M. Milella).

MA-220 is the upper portion of an adult calvarium including the frontal squama, most of both parietals, and planum of the occipital squama (Fig 6, S1 and S2 Videos). The remain does not preserve those diagnostic traits usually evaluated for sex estimation (e.g. glabella, inion, etc.). Its overall robusticity, the apparent prominence of the occipital bone, and the development of the temporal lines may however suggest its classification as a male individual. The degree of closure of cranial sutures [64] points to an age-at-death between 35 and 50 years old. This specimen was the subject of a previous study [39, 65], which linked a series of cut marks along the vault and percussion marks along the periphery to the manipulation of the cranium, and interpreted a circular perforation on the left parietal as a healed trepanation. This analysis, however, did not include a microscopic study of the traces, nor the application of strict taphonomic criteria. We therefore re-evaluated this interesting find according to updated methods and criteria.

Our analysis confirms the presence of percussion marks along the periphery of the calvarium, in the form of repeated notches and nicks along the edge of the cranium, particularly evident on the exocranial surface. The percussion line proceeds along a plane roughly 20 mm above the glabella and the inion and ca. 45 mm below the temporal lines (Fig 6a–6c). In one case, on the exocranial surface of the occipital bone, the impact area is documented by a roughly oval blunt trauma (maximum diameter: 13 mm) with a still-adhering bone flake. In four instances (twice on the frontal bone and on the left and right parietal bones), cracks radiate from the area of impact. Endocranially, the course of the fractures shows an irregular pattern of decortication and deflaking, particularly evident in the probable areas of impact. An old decortication area (maximum width ca. 40 mm) is also present on the right exocranial surface of the frontal bone. In this case, the corresponding endocranial fragment does not show exposure of the diploë. Scrapings, in the form of clusters of subparallel, shallow lesions, are visible on the right and left parietal bones (14 in total). In some cases, the radiating cracks superimpose these scrapings (Figs 5 and 6d).

Additional features of this specimen are two healed fractures and an intravitam perforation of the left parietal bone (Figs 5c and 6e–6g). The two healed fractures are located on the left side of the frontal bone and left parietal, respectively, in both cases at ca. 20 mm from the coronal suture. The one on the frontal bone is the largest, measuring 65 x 10 mm. The trauma on the parietal is smaller and measures 17 x 7 mm. The intravitam perforation is on the posterior side of the left parietal bone and is a circular, funnel-shaped depression of about 22 x16 mm, with reactive and healing processes of the bone tissue (Fig 6e and 6f). The irregularly shaped residual hole measures about 4 mm and it has been alternatively interpreted as the result of a healing trepanation [39] or a dermoid cyst [66], the latter diagnosis being however based only on the study of pictures of the specimen and not on its direct analysis. Fig 6g presents the tomographic image of a detail of the perforation. We can notice the funnel margins, the roughly symmetrical section of the discontinuity, and the sclerotic appearance of the lesion along its margin. Radiocarbon dating of the cranium (Beta-645979) points to 5060 ± 30 BP, therefore to the first depositional phase estimated for the cave (see above).

**Fig 7:**
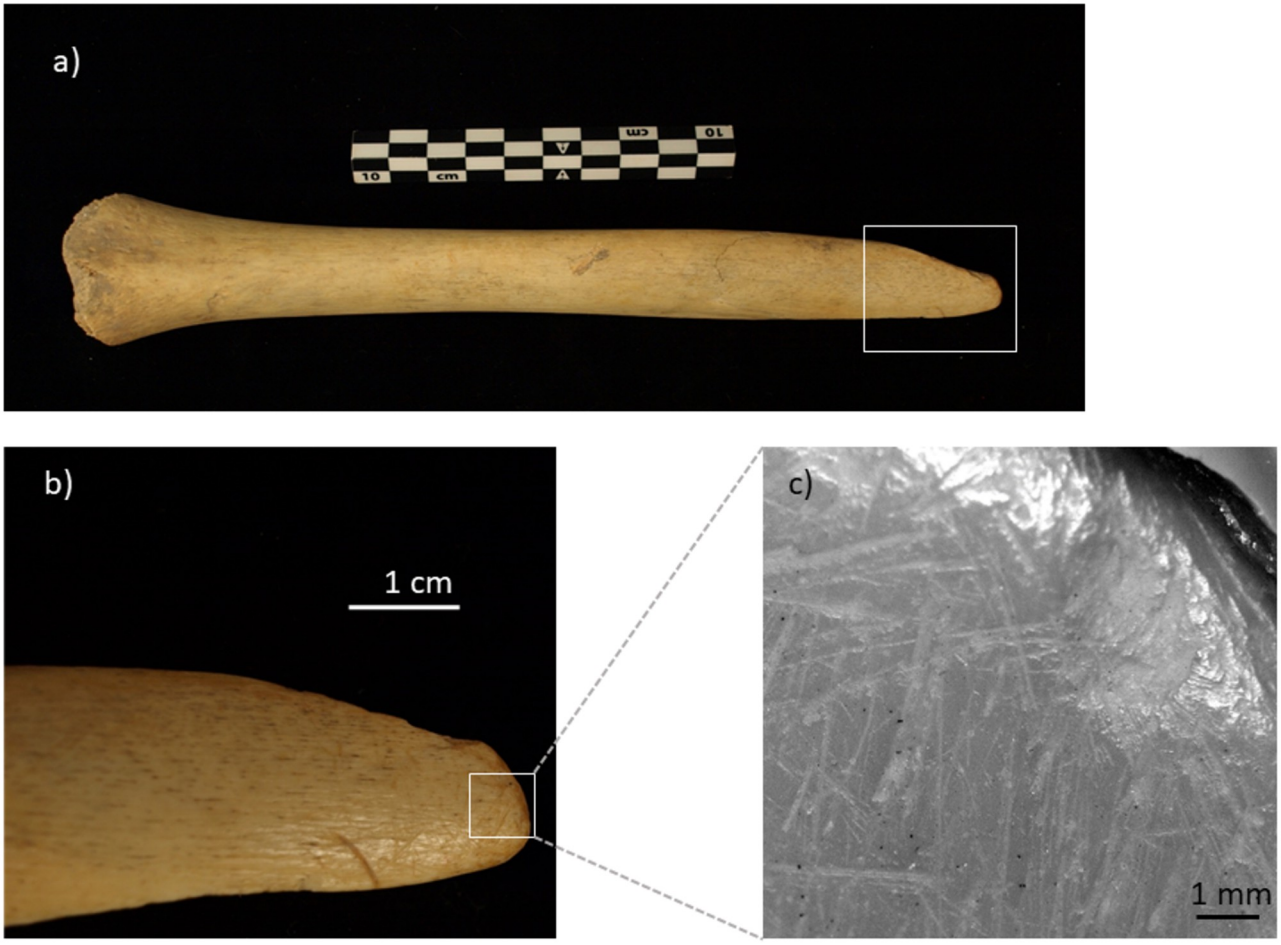
Tibial fragment MA-213-1187: a) overview of the specimen. The rectangle highlights the smoothed proximal end; b) detail of the smoothed proximal end; c) microscopic image of the proximal end (photographs by Z. Laffranchi; digital microscope images captured by J. Brünig).

MA-213-1187 is a partial tibial shaft (length of 243 mm) including most of the mid and distal portion of the diaphysis (Fig 7). The distal epiphysis is missing, and clearly unfused at the time of death. This, in combination with the size of the fragment, would point to its belonging to an older nonadult individual, possibly an adolescent. The fragment ends proximally with an old spiral fracture whose edges show a distinct polished, smooth, and rounded appearance (Fig 7). From the proximal tip of the fragment, for ca. 50 mm, the bone surface is also covered by scratches with no preferential direction. Additional features include a smooth and glossy appearance of the bone along the shaft, and two percussion pits, one on the mid-shaft and another ca. 30 mm from the distal end and still preserving an adhering flake. Interestingly, this is not an isolated case at CM; similar smoothing and polishing were also observed on a fibula fragment (MR18-Z134). Also in these cases, the bone was apparently first broken, and subsequently smoothed at one end through its use as a tool.

## Discussion

With this study, we wanted to address, by means of new data, a traditional research topic of Iberian prehistoric archaeology, i.e. the depositional, chronological, and cultural features of human depositions in caves. We tried to elucidate the tempo and modes of human deposition in a specific, archaeologically well-explored context and applied a three-pronged approach consisting of anthropological, taphonomic, and radiocarbon analyses. Three main patterns emerge from the study: first, radiocarbon data indicate that depositions at CM occurred across at least three millennia and according to three phases. Second, the anthropological study point to a low number of individuals (MNI: 12) and to a lack of specific distribution of the latter in terms of either sex or age-at-death. Third, bone modifications (fresh fractures and alteration of the medullary canal, scraping marks, cut marks and chop marks) hint at heterogeneous treatments of the remains which find intriguing correspondences in other prehistoric contexts from the same region. In the following sections, we discuss the possible cultural meanings of these evidences both contextually and in the broader picture of Prehistoric Andalusia.

### Limits of the study

When evaluating our results it is important to stress the interpretive challenges posed by the peculiar context under study. The first point to address is the actual demographic representativeness of the finds. As mentioned in the introduction, through the years CM has been a target for amateur archaeologists, speleologists, and looters. This likely resulted in the alteration of the original anthropological and archaeological record, both in term of completeness (i.e. retrieval of archaeological and anthropological specimens) and spatial distribution of the finds (i.e. retrieval and discard between different zones). Accordingly, we need to take into account the fact that both the number and spatial distribution of the skeletal elements may be at least partially biased. This has an obvious relevance when discussing the demographic profile of the sample and its possible variability among cave zones. Another point worth considering is the association between taphonomic factors and at least some of the observed skeletal lesions. This issue is particularly pressing since it potentially affects estimates of both timing and mechanism of lesions. Natural caves like CM feature a specific microenvironment, especially in terms of average temperature (colder) and humidity (higher) [67]. Together, these factors are likely to alter both skeletonization and decomposition processes, and, ultimately, the appearance (and estimated timing) of the lesions.

Another aspect worth considering is the possible effect on our estimates of animal activity. At CM, traces of carnivore damage or rodent gnawing were clearly identified on 13 fragments (3% of available skeletal fragments). This result means that, when discussing fragmentation, representation, and spatial distribution of remains we must consider this source of bias. On the other hand, while the presence of these traces complicates our interpretations, their low frequency suggests that animal activity–although present–was somehow limited.

Another potential issue is the possible erroneous attribution of the observed lesions on skeletal remains to anthropic *vs* taphonomic causes. The interpretation of cut and chop marks is relatively more straightforward in this sense thanks to the diagnostic morphological features of these lesions. Conversely, we need to be particularly aware of the possible taphonomic "background noise" when discussing scrapings and fresh fractures. Scrapings may result from sediment attrition [68], whereas fracturing of bones could be the product of rock falls and trampling (both ancient and modern). Combined with the aforementioned effects on bone preservation of a cave’s physical environment, these factors increase the risk of false positives in our records.

We approached the study of the skeletal sample of CM fully aware of these challenges and designed our analytical protocol accordingly. Moreover, the interpretation of lesions as intentional was often helped by contextual criteria. Alterations of the medullary canal, for example, are always associated with fresh fractures, a pattern that, when considered in conjunction with their presence especially on long bones, supports the intentional nature of these features [59, 69]. The anthropogenic nature of scrapings, on the other hand, was not only suggested by their presence on a skeletal element presenting additional traces of manipulation (the "skull-cup" MA-220), but also by their specific anatomical patterning (see below for the detailed interpretation of this find). In our opinion, the biggest issue of this study is therefore not the possible presence of false positives, but rather the likely recording of false negatives, i.e. of lesions conservatively interpreted as taphonomic in origin or excluded due to a dubious appearance. While acknowledging this risk, we also believe that a more parsimonious interpretation is generally preferable to less strict approaches in complex cases like the one at hand.

### Chronology, MNI, and fragmentation

The radiocarbon data from the seven analyzed samples place the deposition of the human remains at CM across at least three millennia, from the late Neolithic to the late Bronze Age and according to at least three distinct depositional phases (Fig 2). This extended time-of-use of the cave would contrast, at least intuitively, with the relatively low MNI estimate (12). Even taking into account that the latter is necessarily conservative, it seems that we are dealing with a sporadic–although continued through time–funerary use of the cave. To better interpret the data from CM, it is useful to compare them with published estimates for other Prehistoric cave contexts from Spain (S7 Table). Such comparisons return a variable scenario, with MNI estimates in some cases similar to those of Marmoles and in other cases largely surpassing it. Relatively more homogenous is the ratio between nonadults and adults, which, with some exceptions, presents similar frequencies although with a slight bias toward adults. At CM and the other sites, nonadults and adults are both represented, although in general adults show higher rates. A calculation of sex ratios on such commingled samples is highly problematic and quite prone to errors. Accordingly, it has been attempted only sporadically and is not considered here. When combining these results (i.e. variable MNI and similar demographic composition), we may postulate (a) different timings and rates in the accumulation of the human remains, and (b) the absence of selection based on the age of the individuals. The first aspect is probably a reflection of various factors, including the heterogeneous timespans represented by each context and the possible variable meaning of cave exploitation by different human groups and cultures. It is worth pointing out that several of these contexts feature traces of manipulation of the human remains. We therefore need to include, among the possible causes for these heterogeneous MNI, the variable aims and meanings of these manipulations (e.g. ritual, functional, and alimentary). The presence of both adults and nonadults across these contexts suggests the absence of rigid selections based on age underlying these depositions and their character of communal, rather than specific, treatment.

Completeness and fragmentation do not appear particularly informative in our case, as the observed patterns seem to reflect the relative fragility of different bones rather than specific post-depositional processes. This is exemplified by the cranium and os coxae, which show relatively low completeness and high fragmentation, as is usually observed in Prehistoric archaeological settings. Smaller and morphologically simpler bones (e.g. metatarsals and metacarpals) or more robust bones (e.g. calcaneus and talus) show opposite patterns. The relatively high fragmentation of femurs and tibia compared with other postcranial bones is however interesting as it appears related to two distinct processes: 1) the easier degradation and fragmentation of the distal and proximal bone regions featuring lower cortical/trabecular ratios (e.g. epiphyses) and 2) the high frequency of fresh fractures, especially on their diaphysis, a result that we discuss more in depth in the following section.

Although neither completeness nor fragmentation show a statistically significant spatial pattern, the number of remains is nonetheless particularly elevated in some cave zones (i.e. II, III, VI, X, XII), conversely dropping in others. This may cautiously be interpreted as the by-product of some sectors of the cave having been destined for the deposition and manipulation of remains, although this interpretation also needs to consider the bias introduced by the mentioned taphonomic factors. It therefore remains an explorative, working hypothesis. In a similar fashion, we can tentatively interpret the specific variability of the Representation Index, which points out clear biases in recovery for some skeletal regions. The low representation of metatarsals and metacarpals, (labile joints–[70, 71]) raises the possibility that individuals were introduced to the cave in a state of partial decomposition. This would be further supported by the low recovery rate of other bones representing labile joints such as hand and foot phalanges (5 bones in total–not included in our analyses), and cervical vertebrae (10 in total *vs* 21 thoracic and 16 lumbar vertebrae). Alternatively, it may be that these bones were simply overlooked and not collected during excavation due to their small size and/or moved and hidden by animals (e.g. rodents). It is worth mentioning however that the recovery of remains from CM was rather detailed, as demonstrated by the presence in the sample of small bone fragments and dental remains. Selective animal damage to hands and feet, although not impossible, is also unlikely based on the relatively scarce frequency of bite and gnawing marks on the remains. Interpreting these data is notoriously difficult [70–72], and these explanations are not mutually exclusive. The preservation and recovery rate of secondarily deposited remains may indeed be affected by animal activity and difficulty in localizing the smallest bones in a cave context. Actualistic and osteoarchaeological data, moreover, suggest a higher-than-expected variability in joint disarticulation patterns, questioning therefore the general applicability of archaeothanatological reconstructions based on intraskeletal differences in joint persistence [73, 74].

### Bone modifications: General patterns

The few cut and chop marks were found mostly on postcranial remains, and their orientation, position, and shape suggest an association to careful actions aimed at disarticulation and/or excarnation. The repeated pattern, distribution on the cranial vault, and shallow depth of the scraping marks on the "skull-cup" MA-220 (see below) indicate an attempt to clean the cranium from residual soft tissues by means of repeated scrapings and the application of a relatively low force. This interpretation would fit the other observed traces of manipulation on this specimen. Fresh fractures are the most frequent type of lesion at CM and are often associated with medullary canal modifications. Combined, this evidence strongly hints at the attempt to access the marrow after crushing the long bone structure, as previously seen in the context of practices well documented for this geographic area and period [69]. Again, it is useful to zoom out and place CM in the larger picture of Prehistoric Andalusia. Neolithic finds from Las Majolicas [75], Cueva del Malamuerzo [76–78], and Cueva del Toro [19] show traces of manipulation remarkably similar to those described here.

At CM, however, cut and scraping marks are comparatively less frequent (1.7% of fragments at CM *vs*. a maximum of 17.5% at Las Majolicas, 30.7% at Malamuerzo, and 10.9% at Cueva de El Toro). While it is geographically and chronologically distant from our case, it is also interesting to point out the frequency difference of cut marks (12.75%) at Gova del Garrofer (Bronze Age of Eastern Spain) [79]. We can propose at least two explanations for these varying frequencies between CM and other, similar sites: (1) the manipulation of the remains took place when they were in a state of advanced decomposition, as also suggested by the poor representation of labile joints. This would have facilitated their disarticulation and reduced the need for cutting soft tissues during their treatment; (2) the manipulation documented by cut marks and fractures are unrelated to each other, and are rather possibly linked to different practices inspired by different purposes. A clear choice between these two options is, based on the available data, not possible.

#### MA-220

The features observed on the vault provide some insights about the timing and breakage dynamics of the cranium. First, the course and direction of the fractures, along the circumference of the cranial vault, strongly hint at their intentional nature. Second, the combined exocranial and endocranial features suggest that the force was applied roughly perpendicular to the exocranial surface, likely by controlled percussions from a blunt object. This interpretation is further supported by a comparison of the CM specimen with published cases of skull-cups from nearby regions [19, 65, 69, 76, 77 among others]. The first known Neolithic skull-cup from Andalusia was discovered in the Cueva de la Carigüela (Píñar, Granada) [65]. It presents incisions along the sagittal line and on the frontal bone, probably resulting from skin removal. It also shows percussion marks consistent with blows to the exocranial surface that were likely responsible for the detachment of the calvarium, as indicated by the perimortem appearance of corresponding fracture lines. The specimen from *Cueva de la Carigüela* has been interpreted as the result of opening the cranium in order to access the brain, in line with the abundant cases of cannibalism archaeologically documented for the Neolithic of Andalusia [69, 76, 77]. Another Neolithic example of skull-cup comes from Cueva de El Toro (Antequera, Málaga) [19]. This find has been interpreted as the final product of a process involving the skinning, percussion, and boiling of a human head. It shows percussion marks along the edge as well as departing cracks quite similar to the scraping marks on the exocranial surface of the CM cranium. Concerning the ultimate cause of the treatment of the cranium at CM, we can consider suggestions previously formulated for similar finds and link the skull-cup to an attempt to access the brain for alimentary purposes [80], or to a procedure aimed at modifying the calvarium for a later use [62]. The second explanation seems supported by the careful retouching observed along the rim of the skull and by the scraping marks on the vault. Of course, we cannot exclude that the retouching of the calvarium followed its opening to access the brain tissue.

The morphology and radiographic appearance of the circular discontinuity on the left parietal bone is not consistent with congenital and developmental defects, secondary malignant tumors, vascular lesions, and metabolic or infectious diseases [81]. This narrows down the possible aetiology to trepanation *vs*. dermoid cyst [cf. 39, 82]. The shape and CT appearance of the lesion weaken the argument for a dermoid cyst, making a healed trepanation the most plausible diagnosis. The practice of trepanation [for a review of archaeological cases see 83] is attested in Andalusia during the Neolithic [66, 84 among others], with most cases involving trepanation by abrasion. In our case, the position, shape, external scarring, and tomographic appearance point to a trepanation by drilling.

#### MA-213-1187

The macroscopic and microscopic morphology of the proximal end of the tibial fragment strongly support the use of the latter as a tool, which would have led to the observed rounding and smoothing. If this hypothesis is correct, then the smooth and glossy appearance of the shaft could be by-products of holding the tool, especially in the case of its repeated use over time. The morphology of the proximal fracture suggests that the tibia was first reduced using a blunt object when the bone was still relatively rich in collagen (not long after death). The two percussion pits on the shaft may indeed be interpreted as preliminary strikes to the bone shaft. It is interesting that a similar modification of human bones is documented at CM by at least two examples (this tibia and the fibula MR18-Z134). This, combined with the previous observation on the skull-cup and lesions observed on the rest of the finds, adds an additional facet to the observed, complex interaction between the living and the dead in the analyzed context. In this respect, it is crucial, however, to ask ourselves if these bones were recognized as human when manipulated or rather confused for faunal remains. This is important, as it may qualify the same action differently, at least from a symbolic perspective. Although we do not have actual data to substantiate clearly one alternative over the other, it is worth considering some aspects: (a) even if for possibly different reasons (e.g. funerary, alimentary, symbolic, or practical), a large portion of human remains at CM show traces of manipulation. The tibia and fibula would therefore fit a pattern of interaction with human remains largely confirmed in the context at hand; (b) the post-mortem manipulation of human remains is a well-known phenomenon during prehistory in Andalusia [69]; (c) it is safe to assume that the prehistoric inhabitants of Andalusia were well-versed in human and animal skeletal anatomy as a result of their funerary practices, which often involved secondary depositions and daily economic interaction with the surrounding natural environment [85].

## Conclusion

As mentioned in the introduction, the funerary exploitation of natural caves is a widespread phenomenon during Prehistory in the Iberian Peninsula and a defining trait of the Neolithic period in Andalusia. An intriguing question therefore pertains to the possible practical and symbolic factors underlying such customs. First, natural caves would have offered a solution to a range of practical issues (e.g. olfactory, visual, and hygienic) related to the disposal of a corpse. However, an exclusively functional interpretation does not seem satisfactory. This is based on the dangers of applying modern Western cultural attitudes to prehistoric societies and considering the complex symbolism characterizing the relationship between the living and the dead for the period under study. This complexity calls for a nuanced interpretation ideally incorporating practical, symbolic, and social elements. On a symbolic level, the perpetual darkness and subterranean placement of caves make them an ideal resting place for community members. Caves are frozen in time, lacking those phenomena usually marking the passing of time (e.g. plant life cycles, seasonal changes in light and temperature, and daily alternation between light and dark); the silence and subterranean position of caves would only add to their liminal connotation. When combined with the presence of human deposition, and especially of collective depositions, these features would also make caves social landmarks and associate them with (admittedly intermingled) concepts such as ancestor veneration, community membership, cultural tradition, and memory [1, 86].

The internal spatial structure of burial chambers (megalithic or hypogeic) and natural caves is reminiscent of the "socially active" houses of the living, conferring to the community of the dead the social role of "ancestors" [87]. Such cultural elements may vary, but the proximity of and interaction with the physical remains of the deceased seems a rather focal aspect of a system aimed at maintaining and reproducing the social order [88].

In this sense, the practices documented at CM fit well with those widely documented in large areas of the Iberian Peninsula during Late Prehistory, and suggest the possibility of shared ideologies where the human body becomes central in maintaining the collective memory of a community [88, 89].

This leads us to one of the central aspects of this study: the ultimate purpose of the manipulation observed at CM over time. As already mentioned, whereas patterns of bone fractures would support cannibalistic practices, the scarcity of cut marks makes this explanation not fully satisfying. Disarticulation and defleshing would lead to a high frequency of cut marks on the skeletal remains of consumed human and nonhuman individuals and, especially, to their distribution consistent with butchering practices [45, 90]. A possible explanation for such counterintuitive results may come from the Neolithic finds of Scaloria Cave (Southern Italy) [11]. Here, skeletal remains present fresh bone breakages as well as fine cut marks (found on 5.5% of remains) consistent with the removal of residual soft tissues, but not with butchering. To the study’s authors, this feature suggested their association to secondary practices involving the retrieval of body parts from the area surrounding the cave, the cleaning and breakage of the bones, and their casual abandonment on the cave floor [11]. This interpretation of the finds from Scaloria suggests the possibility of a similar scenario for CM, namely a secondary treatment of the remains, as suggested earlier based on joint preservation. This would have included their selection, modification (possibly including consumption of marrow), and abandonment in the cave.

Finally, it is important to stress that discussions of caves as spaces reserved for collective funerary deposits should not be dissociated from those of the "constructed" and megalithic collective burials, passage graves, and chambered tombs found in most of Western Europe. In this sense, we have an increasingly large body of ^14^C dates that place southern Iberia as an early focus in the development of this phenomenon from c. 4000 cal. BCE [8, 22, 91, 92]. This fits chronologically with the first depositional phase documented at CM. The possibility that “natural” burial caves were perceived differently from the “constructed” megalithic phenomenon itself is a pertinent but difficult question, and one that most likely has different answers considering the temporal and spatial-regional dimension of a heterogeneous and complex phenomenon.

## Supporting information

S1 Fig Absolute (N) and relative (%) frequency of fragments by skeletal element. The cranium is considered as a single element.
(TIF)

S2 Fig Representation index by side and skeletal element for adults and nonadults. (TIF)

S3 Fig Percentage of each MNE calculated for each skeletal element on total skeletal MNE by cave zone. (TIF)

S4 Fig Variability of lesion profiles on a selected subsample of cut marks. Size is standardized in order to facilitate comparison. (a) cut mark on the right femur MR18-Z165, anterior side of the neck surface; (b) scraping mark on the "skull cup" MA-220 right parietal bone; (c) cut mark on left hemi mandible MR-18-Z124 inferior mandibular border; (d) incision on the "skull-cup" MA-220 left parietal bone; (e) scraping mark on the "skull cup" MA-220 left parietal bone; (f) cut mark on right femur MR18-Z165 anterior side of the neck surface. Note in all cases the asymmetric borders, shallow profiles, and relatively broad width of the lesions.
(TIF)

S1 Table Results of radiocarbon dating of the seven bone samples. R: right, L: left.
(XLSX)

S2 Table Dataset used in this study. The landmark numbers followed by a "t" refer to those scored on teeth. For the definition of each landmark see Mack et al. [44]. M: molar; P: premolar; I: permanent incisor; i: deciduous incisor; C1-7: cervical vertebrae; T1-12: thoracic vertebrae; L1-5: lumbar vertebrae; S: sacral vertebrae; L: left; R: right.
(XLSX)

S3 Table Total and zone-specific Percentage Completeness. Roman numerals indicate cave zones. Empty cells indicate cave zones with no remains of a specific element.
(XLSX)

S4 Table Total and zone-specific Fragmentation Index values. Roman numerals indicate cave zones. Empty cells indicate cave zones with no remains of a specific element.
(XLSX)

S5 Table Minimum number of elements (MNE) and minimum number of individuals (MNI) with and without lesions. f: fresh fractures; c: cut marks; ch: chop marks; malt: medullary cavity alteration; L: left; R: right.
(XLSX)

S6 Table Absolute (n) and relative (%) frequency of fragments with lesions by skeletal element. The table also includes fragments with no landmarks available and does not consider sides separately. M: molar; P: premolar; I: permanent incisor; i: deciduous incisor; C1-7: cervical vertebrae.
(XLSX)

S7 Table Summary of Spanish cave contexts [16, 19, 24, 93–107] with human remains. (XLSX)

S1 Video Photogrammetric 3D model of MA-220 (exocranial view). Model and animation prepared by M. Milella.
(MP4)

S2 Video Photogrammetric 3D model of MA-220 (endocranial view). Model and animation prepared by M. Milella.
(MP4)
