# Supplementary figures and images for: As above, so below: Deposition, modification, and reutilization of human remains at Marmoles cave (Cueva de los Marmoles: Southern Spain, 4000–1000 cal. BCE)
Source: PLoS One. 2023 Sep 20;18(9):e0291152. doi: 10.1371/journal.pone.0291152 (PMC10511113; doi:10.1371/journal.pone.0291152)

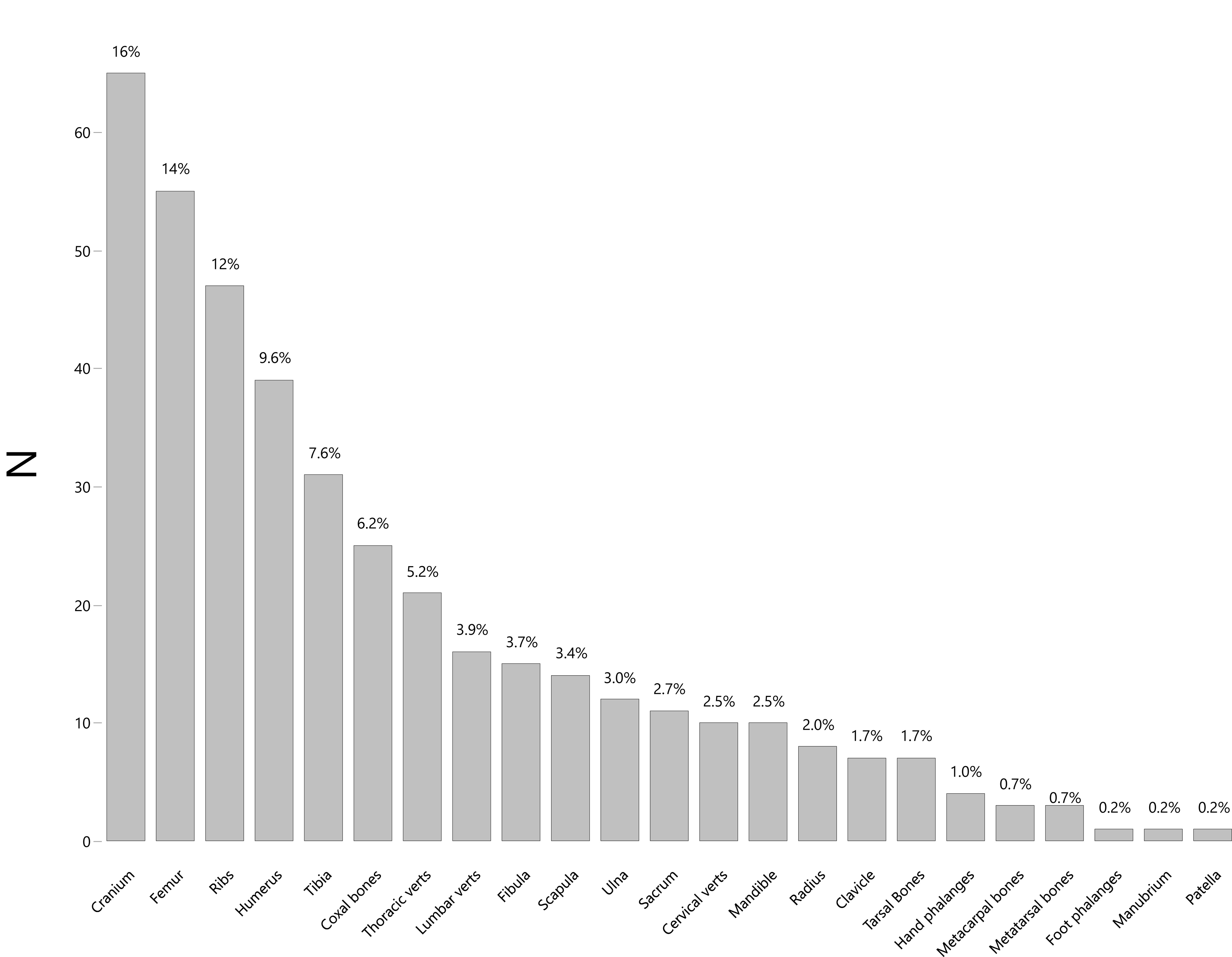

Supplement: S1 Fig — The cranium is considered as a single element. (TIF) [file pone.0291152.s001.tif]

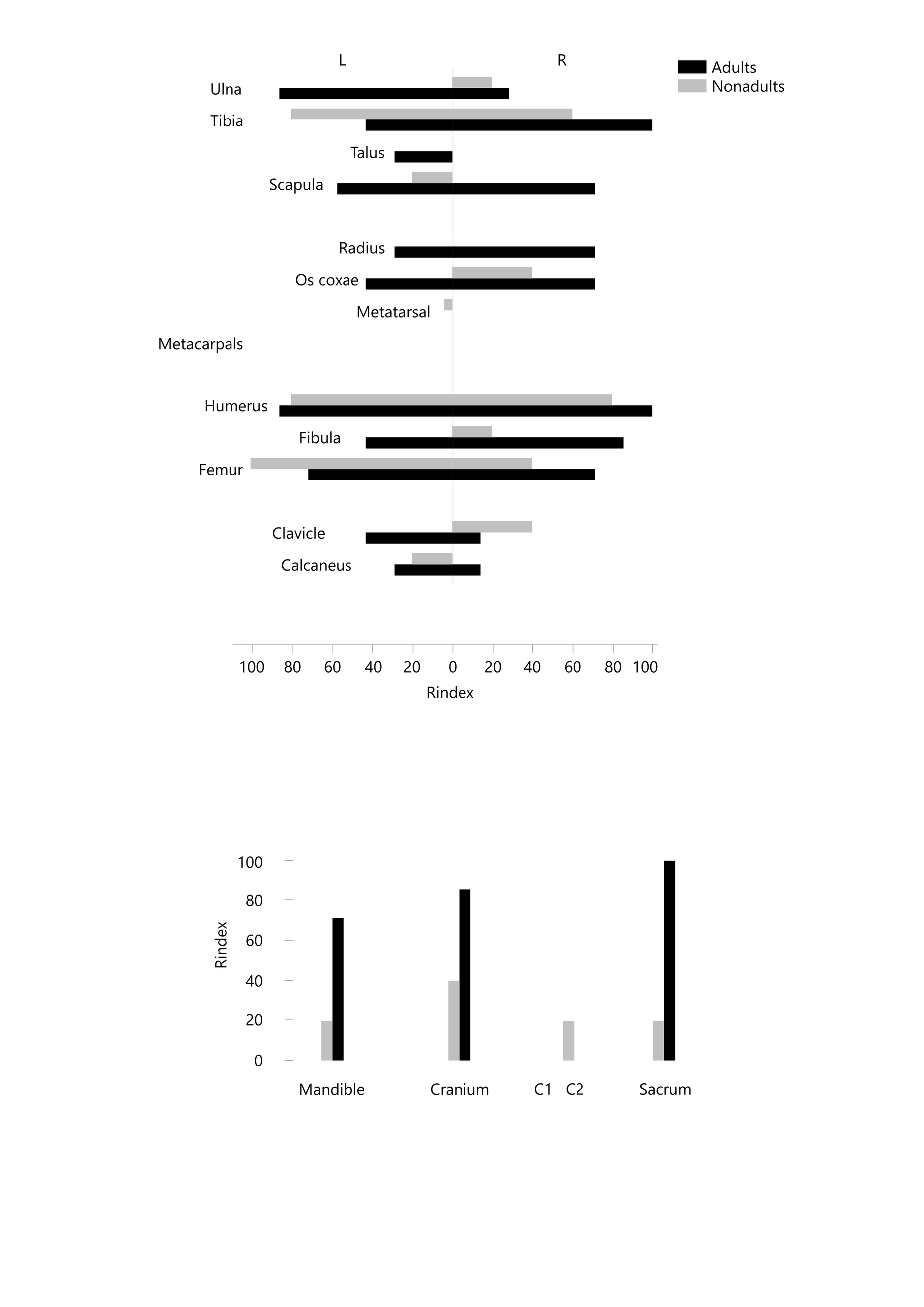

Supplement: S2 Fig — (TIF) [file pone.0291152.s002.tif]

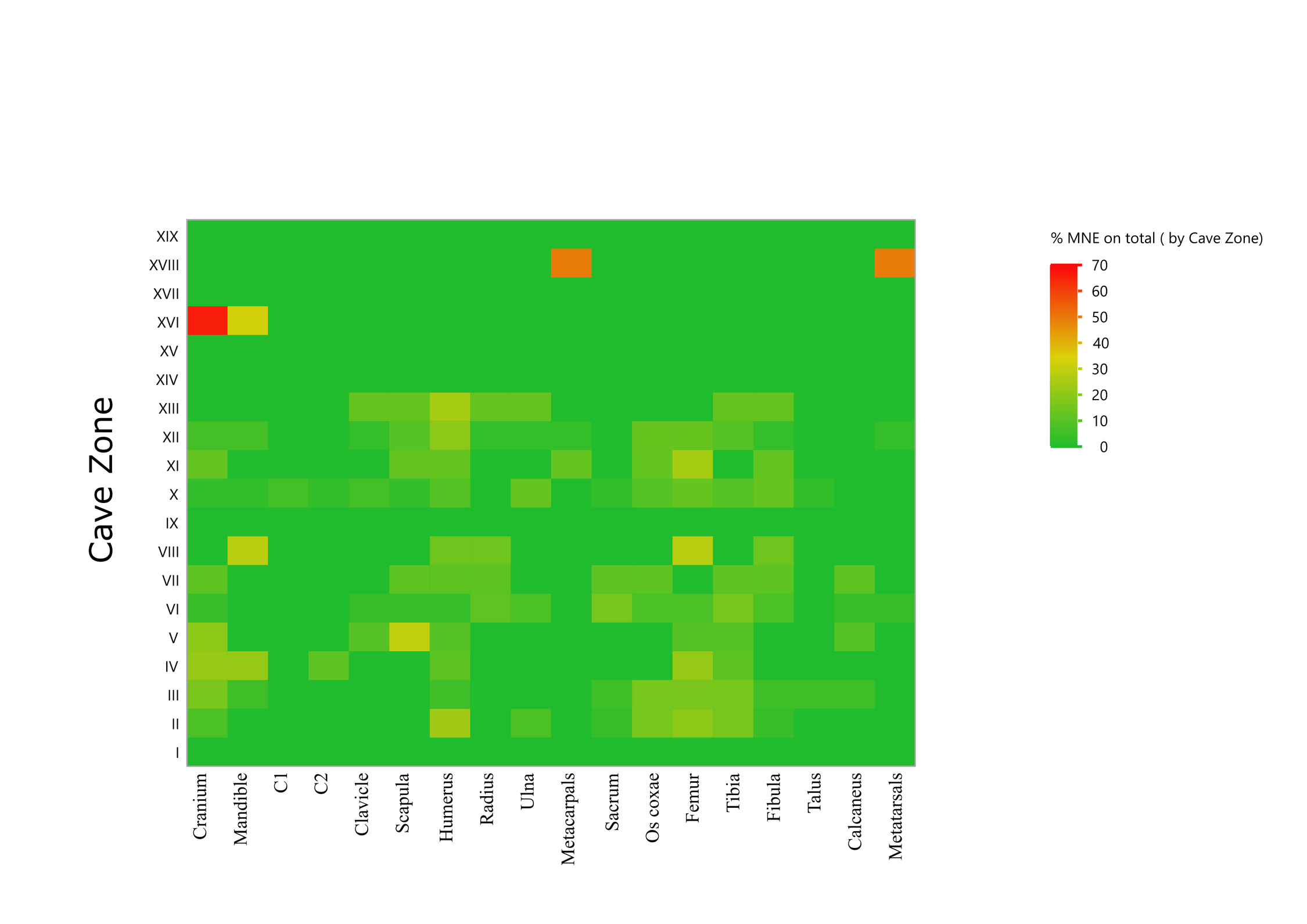

Supplement: S3 Fig — (TIF) [file pone.0291152.s003.tif]

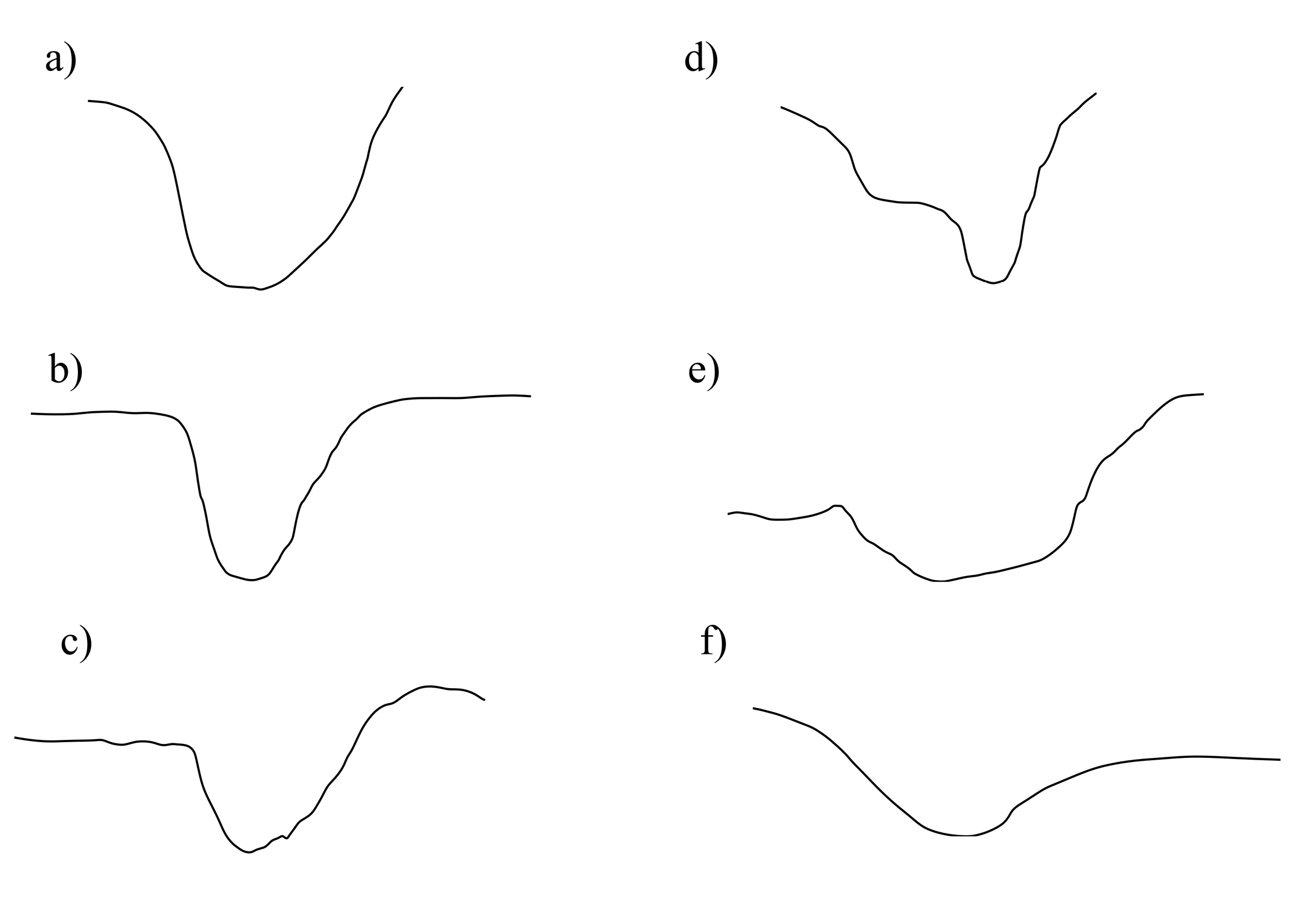

Supplement: S4 Fig — Size is standardized in order to facilitate comparison. (a) cut mark on the right femur MR18-Z165, anterior side of the neck surface; (b) scraping mark on the "skull cup" MA-220 right parietal bone; (c) cut mark on left hemi mandible MR-18-Z124 inferior mandibular border; (d) incision on the "skull-cup" MA-220 left parietal bone; (e) scraping mark on the "skull cup" MA-220 left parietal bone; (f) cut mark on right femur MR18-Z165 anterior side of the neck surface. Note in all cases the asymmetric borders, shallow profiles, and relatively broad width of the lesions. (TIF) [file pone.0291152.s004.tif]
